# Supplementary material for: The Influence of Coumestrol on Sphingolipid Signaling Pathway and Insulin Resistance Development in Primary Rat Hepatocytes
Source: Biomolecules. 2021 Feb 12;11(2):268. doi: 10.3390/biom11020268 (PMC7918648; doi:10.3390/biom11020268)
Supplement: Supplementary file 1 [file biomolecules-11-00268-s001.pdf]

Fig. 1

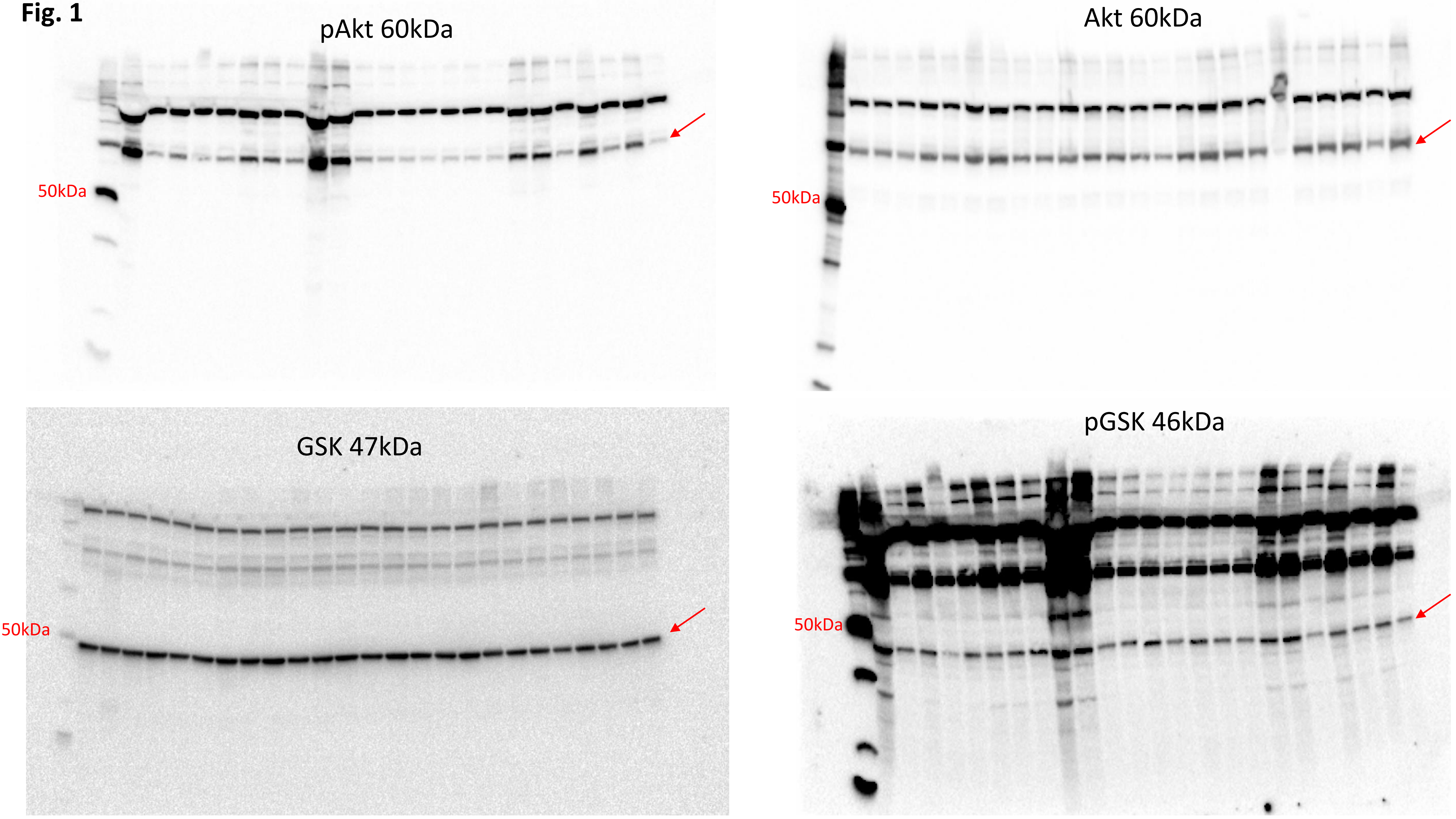
pAkt 60kDa
50kDa
Akt 60kDa
50kDa
GSK 47kDa
50kDa
pGSK 46kDa
50kDa

Fig. 2

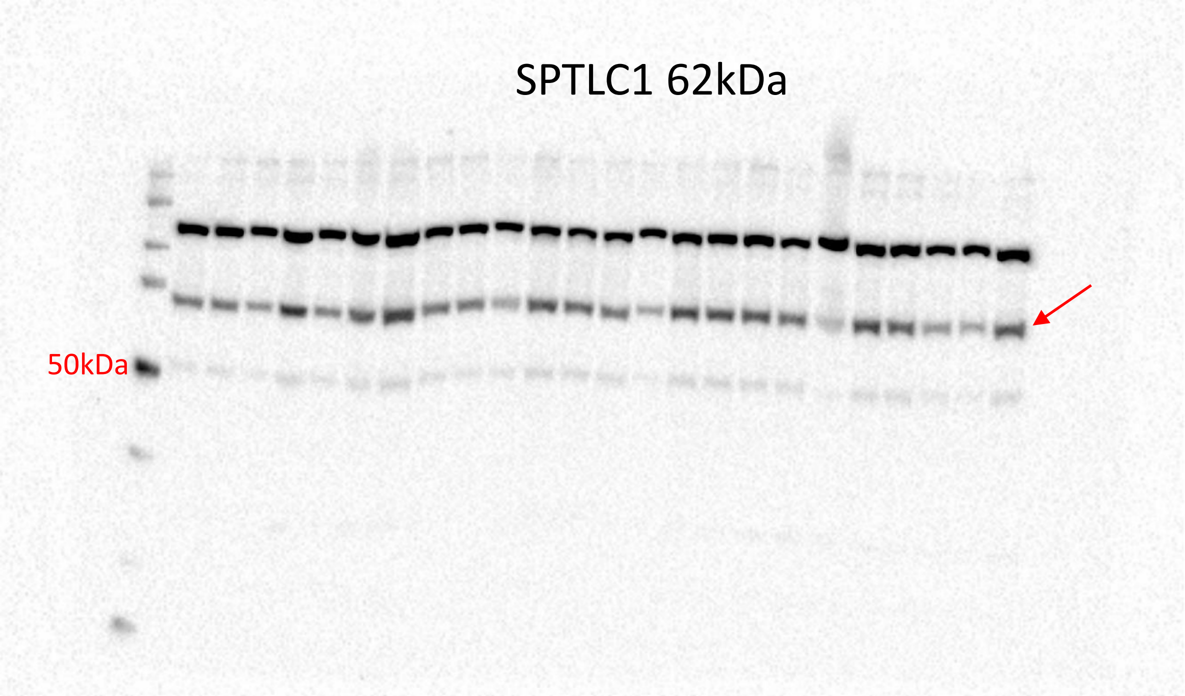

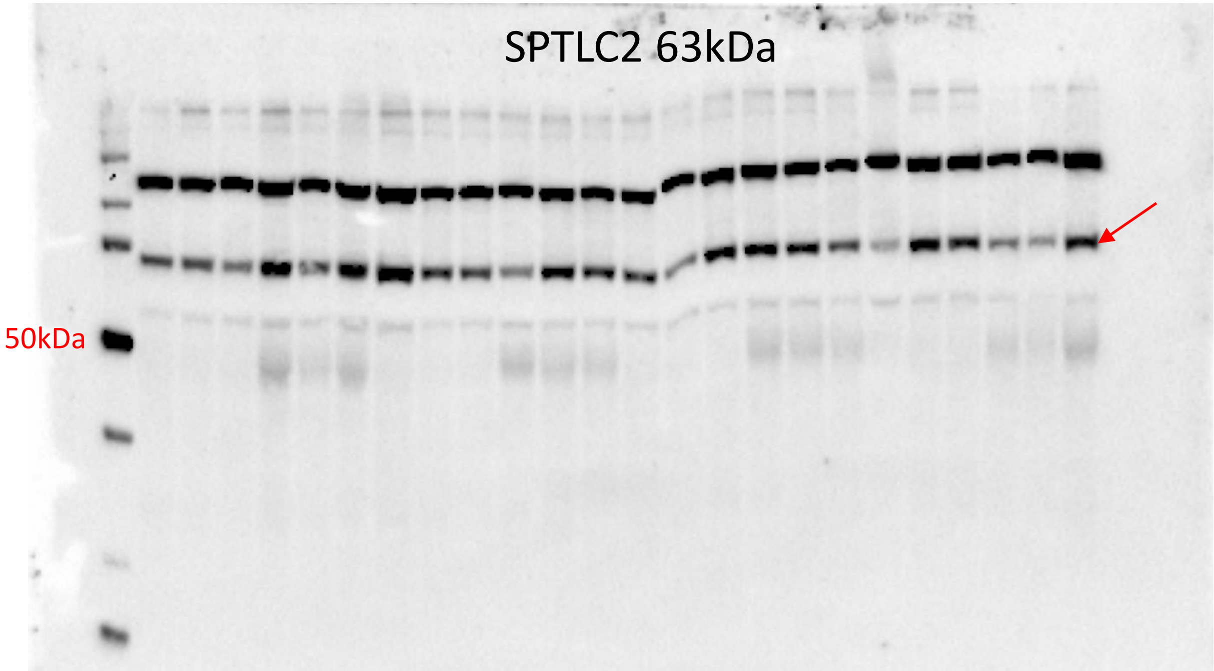

Fig. 3

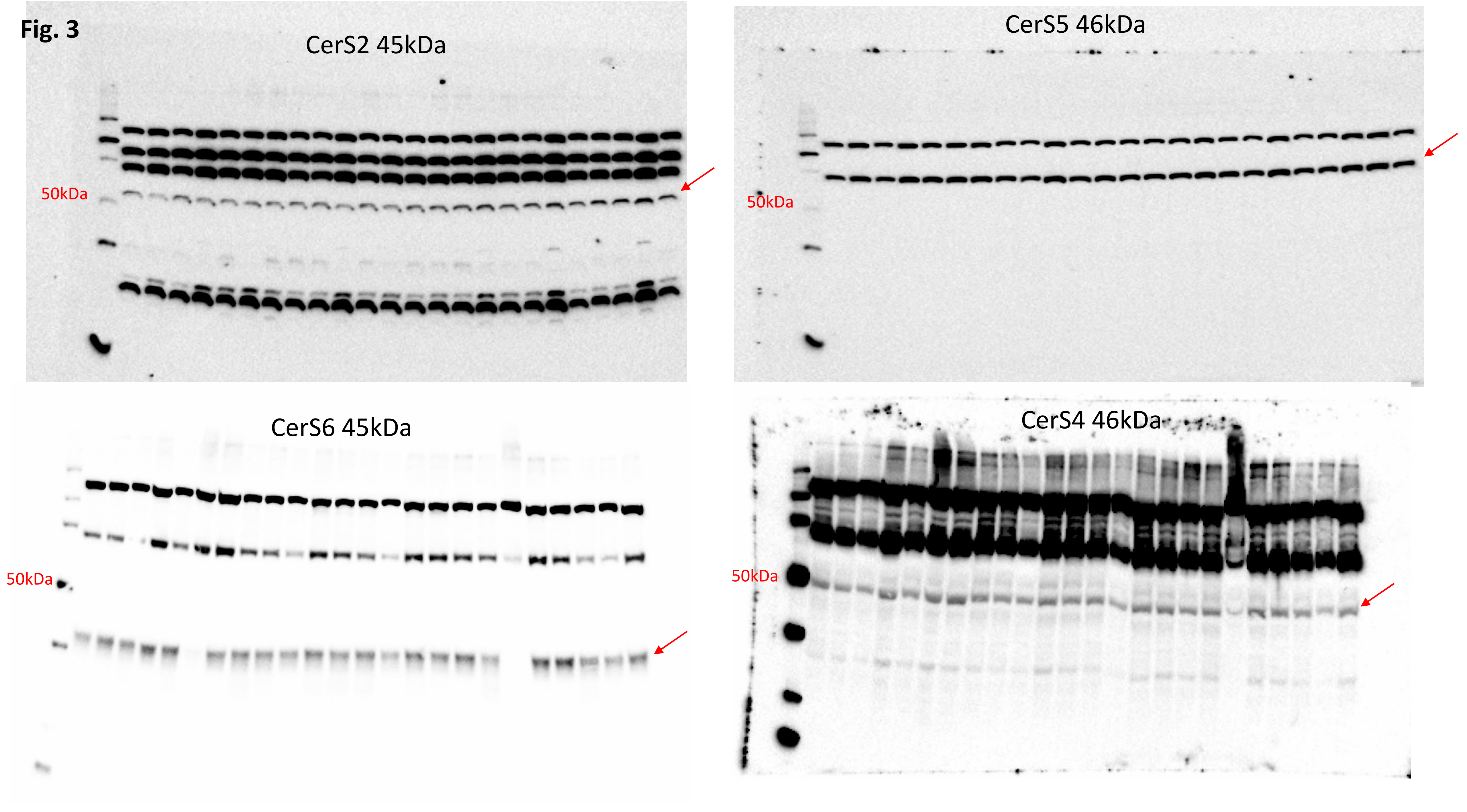
CerS2 45kDa
50kDa
CerS5 46kDa
50kDa
CerS6 45kDa
50kDa
CerS4 46kDa
50kDa

Fig. 4

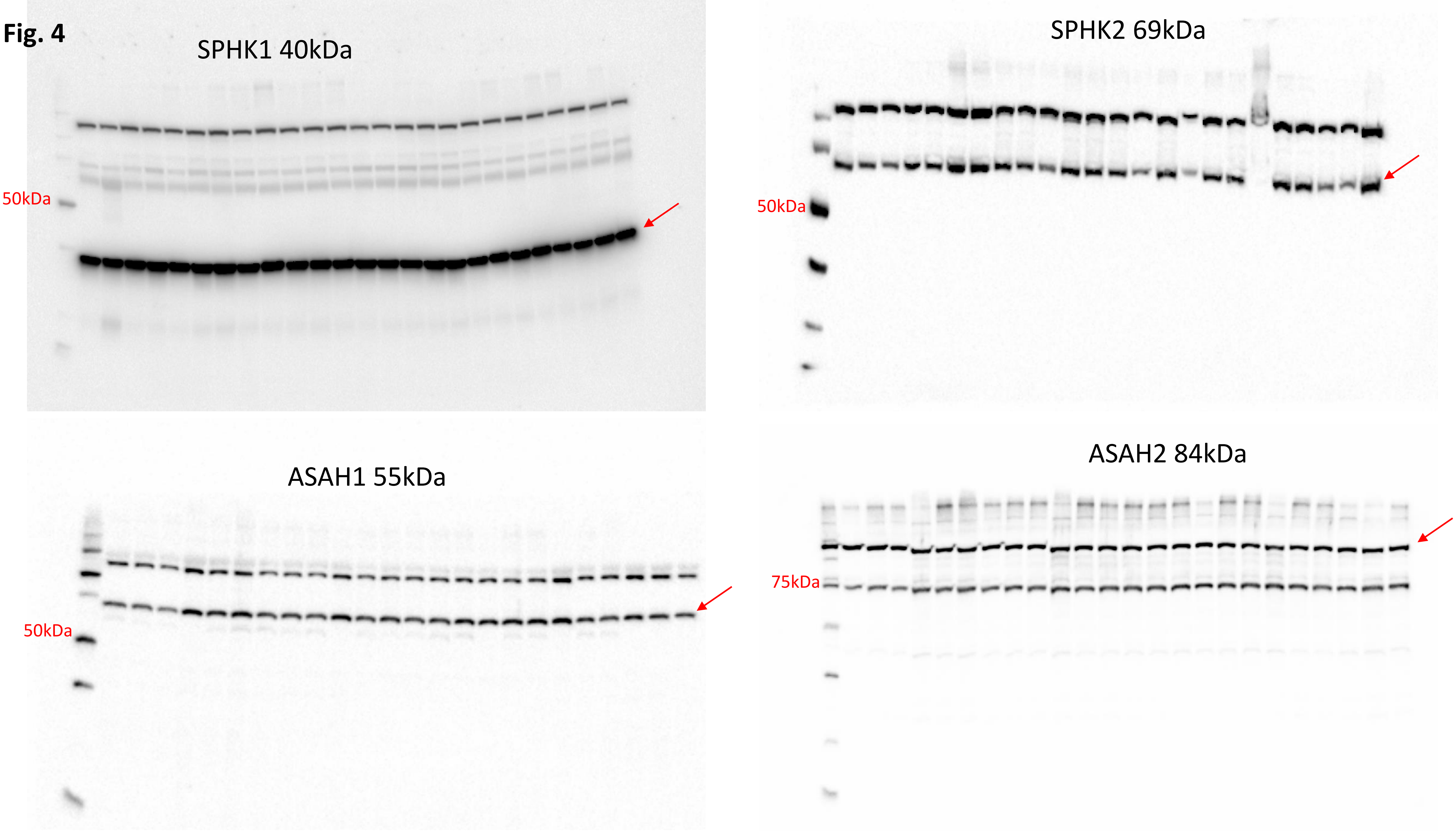
SPHK1 40kDa
50kDa
SPHK2 69kDa
50kDa
ASAH1 55kDa
50kDa
ASAH2 84kDa
75kDa

Fig. 5

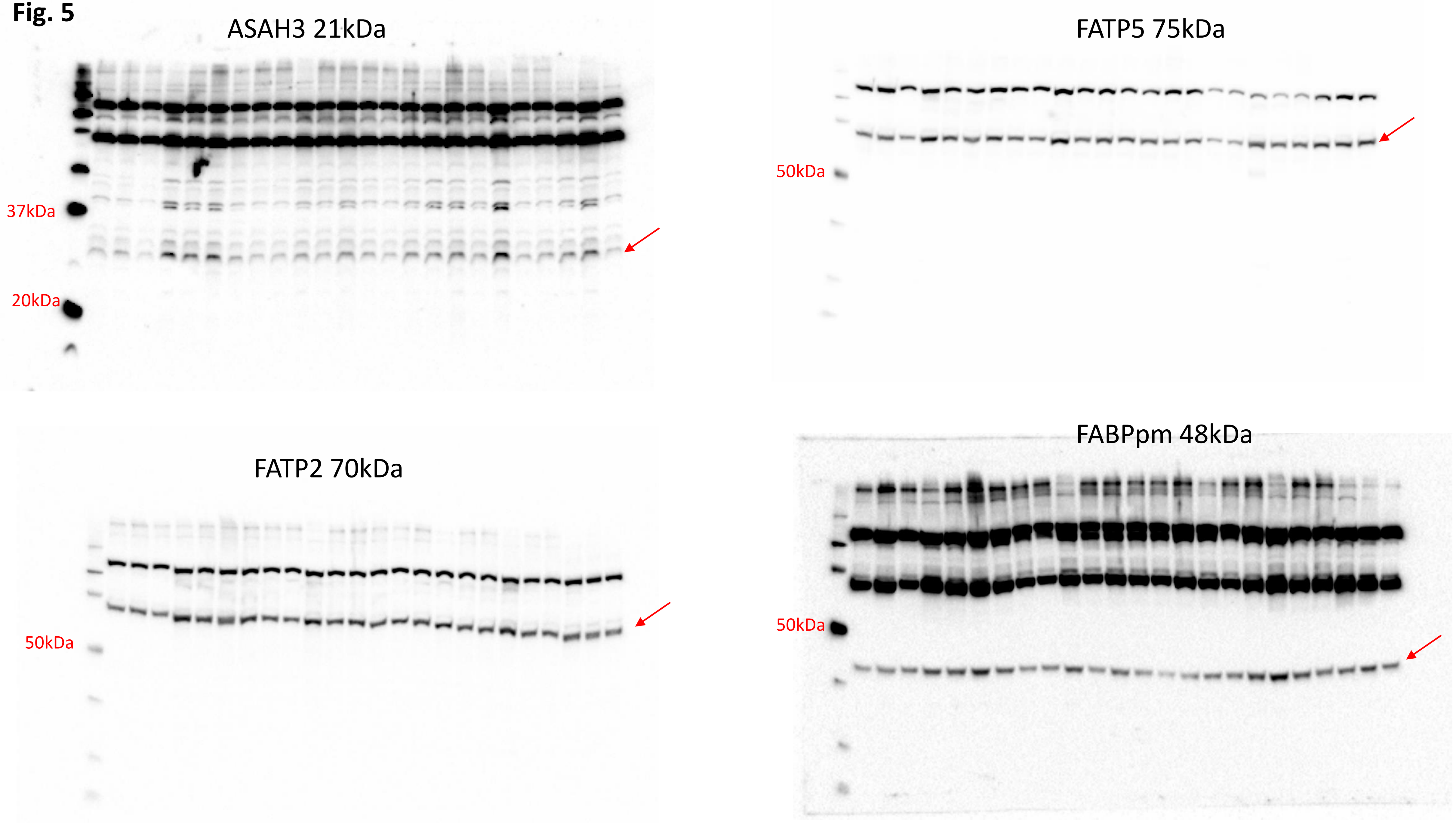
ASAH3 21kDa
37kDa
20kDa
FATP5 75kDa
50kDa
FATP2 70kDa
50kDa
FABPpm 48kDa
50kDa

Fig. 6

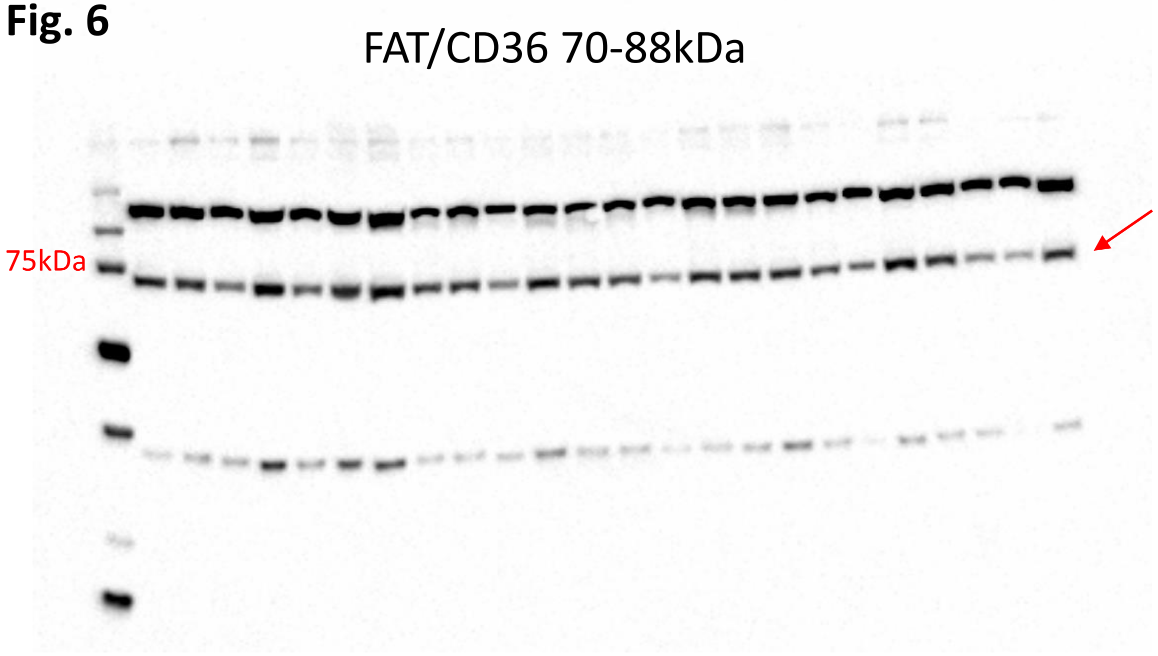

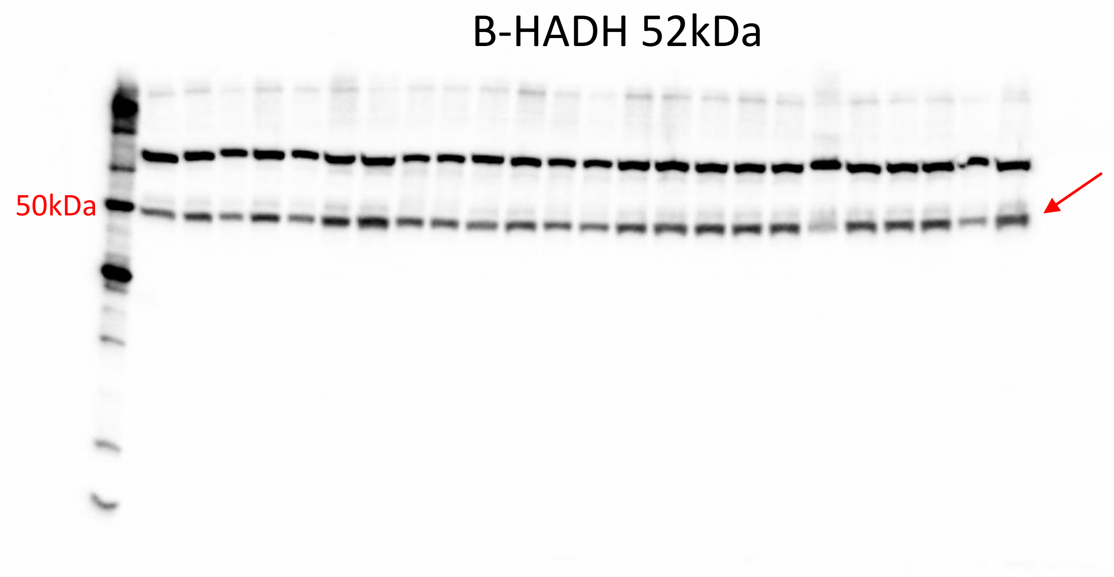

Fig. 7

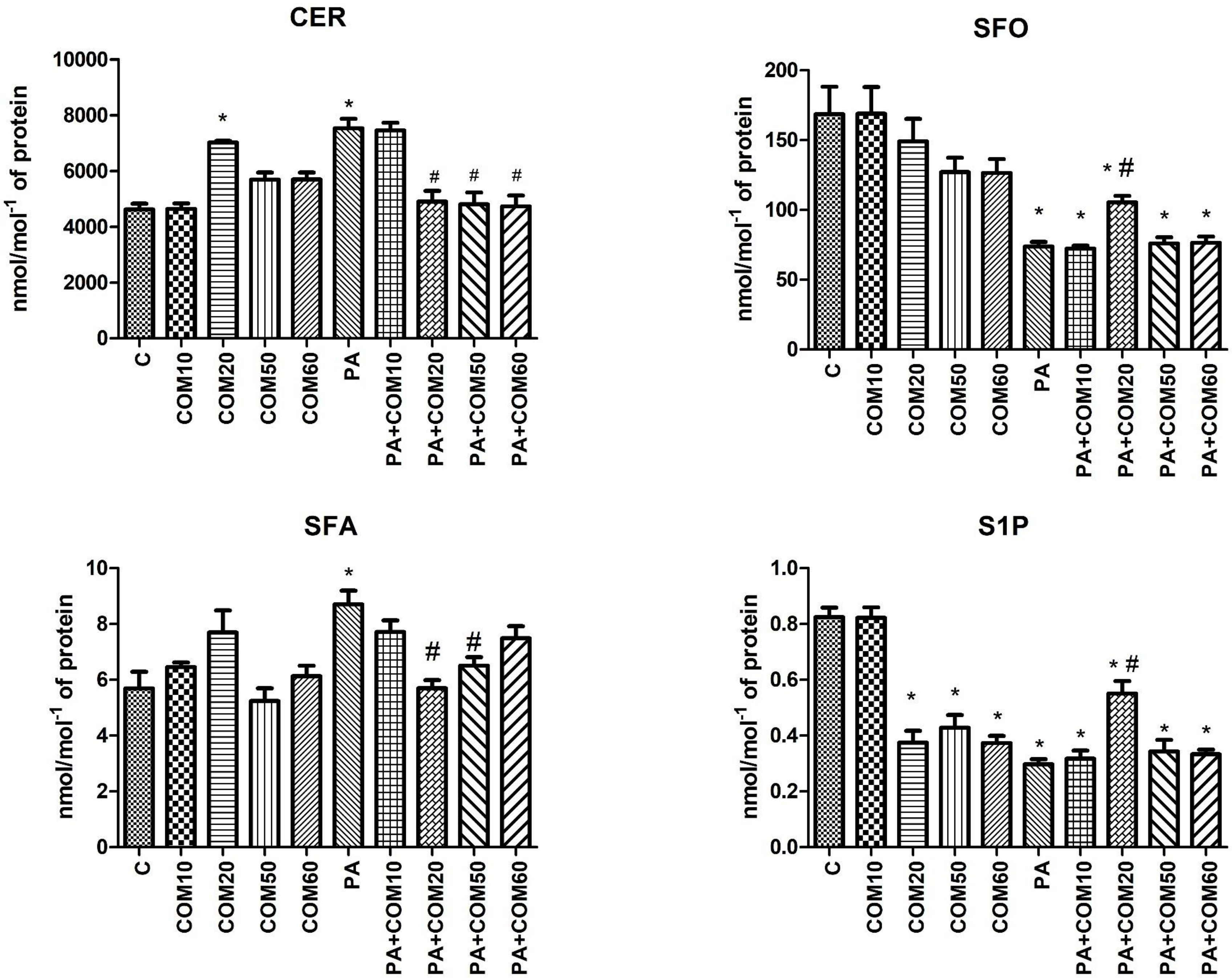
CER
SFO
SFA
S1P
nmol/mol-1 of protein
C
COM10
COM20
COM50
COM60
PA
PA+COM10
PA+COM20
PA+COM50
PA+COM60
